# Characterization of the RnfB and RnfG Subunits of the Rnf Complex from the Archaeon *Methanosarcina acetivorans*

**DOI:** 10.1371/journal.pone.0097966

**Published:** 2014-05-16

**Authors:** Suharti Suharti, Mingyu Wang, Simon de Vries, James G. Ferry

**Affiliations:** 1 Department of Biochemistry and Molecular Biology, The Pennsylvania State University, University Park, Pennsylvania, United States of America; 2 Department of Biotechnology, Delft University of Technology, Delft, The Netherlands; University of Florida, United States of America

## Abstract

Rnf complexes are redox-driven ion pumps identified in diverse species from the domains *Bacteria* and *Archaea*, biochemical characterizations of which are reported for two species from the domain *Bacteria*. Here, we present characterizations of the redox-active subunits RnfG and RnfB from the Rnf complex of *Methanosarcina acetivorans*, an acetate-utilizing methane-producing species from the domain *Archaea*. The purified RnfG subunit produced in *Escherichia coli* fluoresced in SDS-PAGE gels under UV illumination and showed a UV-visible spectrum typical of flavoproteins. The Thr166Gly variant of RnfG was colorless and failed to fluoresce under UV illumination confirming a role for Thr166 in binding FMN. Redox titration of holo-RnfG revealed a midpoint potential of −129 mV for FMN with n = 2. The overproduced RnfG was primarily localized to the membrane of *E. coli* and the sequence contained a transmembrane helix. A topological analysis combining reporter protein fusion and computer predictions indicated that the C-terminal domain containing FMN is located on the outer aspect of the cytoplasmic membrane. The purified RnfB subunit produced in *E. coli* showed a UV-visible spectrum typical of iron-sulfur proteins. The EPR spectra of reduced RnfB featured a broad spectral shape with *g* values (2.06, 1.94, 1.90, 1.88) characteristic of magnetically coupled 3Fe-4S and 4Fe-4S clusters in close agreement with the iron and acid-labile sulfur content. The ferredoxin specific to the aceticlastic pathway served as an electron donor to RnfB suggesting this subunit is the entry point of electrons to the Rnf complex. The results advance an understanding of the organization and biochemical properties of the Rnf complex and lay a foundation for further understanding the overall mechanism in the pathway of methane formation from acetate.

## Introduction

**Figure 1:**
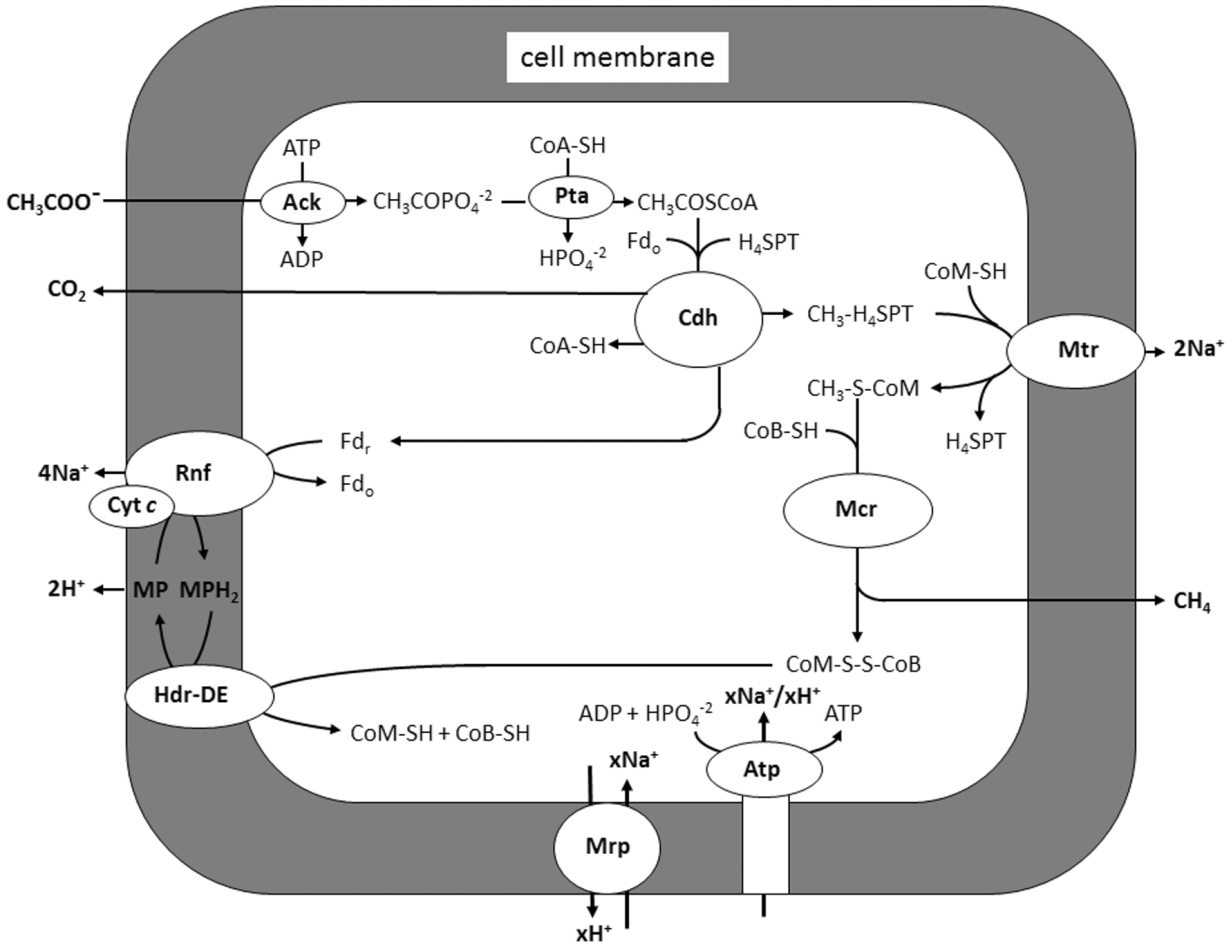
Pathway of aceticlastic methanogenesis proposed for *M. acetivorans*. Ack, acetate kinase; Pta, phosphotransacetylase; CoA-SH, coenzyme A; H_4_SPT, tetrahydrosarcinapterin; Fd_r_, reduced ferredoxin; Fd_o_, oxidized ferredoxin; Cdh, CO dehydrogenase/acetyl-CoA synthase; CoM-SH, coenzyme M; Mtr, methyl-H_4_SPt:CoM-SH methyltransferase; CoB-SH, coenzyme B; MP, methanophenazine; Hdr-DE, heterodisulfide reductase; Rnf, Rnf complex; Mrp, Mrp complex; Atp, ATP synthase. Modified from [17].

Acetate is the major source of biological methane in nature. Only two genera (*Methanosarcina* and *Methanosaeta*) of acetate-utilizing methane-producing microbes are known of which *Methanosarcina* species have been researched to the greatest extent. Although the pathway for conversion of the methyl group of acetate to methane is well documented [1], less is understood concerning electron transport recently reviewed [2]. The great majority of acetate-utilizing *Methanosarcina* species are unable to metabolize H_2_ [3] of which *Methanosarcina acetivorans* is a model. Exceptions to this majority are *Methanosarcina barkeri* and *Methanosarcina mazei* which are dependent on the production and consumption of H_2_ for electron transport coupled to generation of a proton gradient driving ATP synthesis [2]. The acetate-to-methane pathway in *M. acetivorans* (Fig. 1) begins with activation of acetate to acetyl-CoA, the substrate for the CO dehydrogenase/acetyl-CoA synthase (Cdh) complex. Cdh cleaves the C-C and C-S bonds yielding HS-CoA, a methyl group which Cdh transfers to tetrahydrosarcinapterin (THSPt) yielding CH_3_-THSPT, and a carbonyl group that Cdh oxidizes to CO_2_ with transfer of the electrons to ferredoxin (Fd). The CH_3_-THSPT produced by Cdh is transferred to coenzyme M (HS-CoM) catalyzed by a membrane complex (Mtr). The exothermic reaction producing CH_3_-S-CoM is coupled to generation of a Na^+^ gradient with the potential to drive ATP synthesis. Methylreductase (Mcr) catalyzes reduction of the methyl group of CH_3_-S-CoM to methane with electrons donated by the sulfur atoms of coenzyme B (HS-CoB) and CH_3_-S-CoM. The heterodisulfide CoM-S-S-CoB, a product of the CH_3_-S-CoM demethylation reaction, is reduced to the active sulfhydryl forms of the cofactors by heterodisulfide reductase (HdrDE). The two electrons required for this reduction are derived from oxidation of the carbonyl group of acetate catalyzed by Cdh for which Fd is the electron acceptor.

Transfer of electrons from Fd to HdrDE by *M. acetivorans* involves a membrane-bound electron transfer chain remarkably distinct from H_2_-utilizing *M. barkeri* and *M. mazei*. In *M. barkeri* and *M. mazei*, Fd donates electrons to a hydrogenase complex (Ech) that produces H_2_ and generates a proton gradient for ATP synthesis [4]–[7]. A hypothesis has been advanced wherein H_2_ is re-oxidized by another membrane-bound hydrogenase (Vho) depositing protons outside the cell membrane and transferring electrons to methanophenazine (MP) functionally equivalent to quinones [8]. Finally, reduced MP donates electrons to HdrDE reducing CoM-S-S-CoB to HS-CoM and HS-CoB accompanied by translocation of protons which further contributes to ATP synthesis.

An electron transport chain is proposed for *M. acetivorans*, the only non-H_2_-metabolizing *Methanosarcina* species for which the genome is sequenced [9]. The genome does not encode Ech hydrogenase [10], further excluding H_2_ in electron transport. In contrast to *M. barkeri* and *M. mazei*, *M. acetivorans* synthesizes an eight-subunit membrane-bound complex (Rnf) with deduced sequence identity to the six-subunit Rnf (R *hodobacter n*itrogen *f*ixation) complex of *Rhodobacter capsulatus* from the domain *Bacteria* where the complex is proposed to function in reverse electron transport oxidizing NADH and reducing Fd that supplies electrons to nitrogenase [11]. Subunits of the *M. acetivorans* Rnf complex are elevated 10-fold in acetate- *versus* methanol-grown cells consistent with a role in acetotrophic growth [11], [12]. Furthermore, it is reported that a Δ*MA0658-0665* mutant of *M. acetivorans* fails to grow with acetate as the sole substrate [13]. The eight genes (MA0657-64) encoding the Rnf complex are co-transcribed in a unit which contains genes encoding a multi-heme cytochrome *c* (MA0658), and a hypothetical membrane-integral protein (MA0665) not present in Rnf complexes from the domain *Bacteria* [11]. The protein encoded by MA0665 has no recognizable motifs that would indicate the presence of redox active cofactors and is proposed to anchor cytochrome *c* to the membrane [11]. The cytochrome *c* is synthesized at high levels in acetate-grown cells where it dominates the UV-visible spectrum of purified membranes [11]. Reduction of purified membranes with Fd from acetate-grown cells leads to reduction of the cytochrome *c* that is re-oxidized by the addition of either CoM-S-S-CoB or 2-hydoxyphenazine, an analog of MP [9]. Reduced 2-hydoxyphenazine is re-oxidized by membranes dependent on the addition of CoM-S-S-CoB. The results suggest electron transport from cytochrome *c* to MP that donates electrons to HdrDE. It is postulated that MP is a potential coupling site for the translocation of protons analogous to the role of MP in the pathway of methanol conversion to methane [2], [9]. The combined evidence supports a H_2_-independent membrane-bound electron transport chain originating with Fd and culminating with reduction of CoM-S-S-CoB involving the Rnf complex, cytochrome *c* and MP. More recently, it was reported that inverted membrane vesicles of *M. acetivorans* catalyze Na^+^ transport coupled to the oxidation of Fd and reduction of CoM-S-S-CoB [14]. It was further reported that a Δ*rnf* mutant is unable to grow with acetate and that Na^+^ transport coupled to Fd:CoM-S-S-CoB oxidoreductase activity of membranes is abolished [14], a result which supports a role for the Rnf complex in generation of a Na^+^ gradient supplementing the Na^+^ gradient generated by Mtr. Thus, it is anticipated that both Na^+^ and H^+^ gradients are generated during acetate-dependent growth of *M. acetivorans*. This conjecture is consistent with the concurrently coupled translocation of both Na^+^ and H^+^ by the ATP synthase of *M. acetivorans* [15], [16]. It was recently proposed that a multi-subunit Na^+^/H^+^ antiporter (Mrp) functions to adjust the ratio of Na^+^ and H^+^ gradients to maximize the efficiency of the ATP synthase [17].

Since the initial discovery in *R. capsulatus*, Rnf homologs have been discovered in diverse species from the domains *Bacteria* and *Archaea*, many of which are unable to fix nitrogen and with diverse metabolisms suggesting diverse functions for Rnf complexes [18]–[34]. In addition to providing reductant for nitrogenase, proposed functions include the generation of ion gradients coupled to the reduction of NAD^+^ with Fd during caffeate respiration [18], [25], [28], [29], acetogenesis [34] and the ethanol-acetate fermentation [19]. An Rnf complex is postulated to function in ion gradient-driven reversed electron transport from NADH to Fd during the syntrophic metabolism of fatty acids and benzoate [20], [30]. Rnf complexes are abundant in sulfate reducing species, although a function has not been proposed [31]. Genomic sequencing has revealed genes encoding Rnf complexes in a wide array of diverse microorganisms. Although Rnf complexes are widely distributed and function in diverse metabolisms, only two biochemical investigations are reported and neither from methanogens or the domain *Archaea* [24], [35]. Clearly, organisms with diverse metabolisms are expected to have evolved specialized biochemical properties of Rnf complexes. The complex from *M. acetivorans* stands apart from characterized Rnf complexes based on its function in methanogenesis, inability to reduce NADH or NADPH [9], [14], reduction of the electron acceptor MP and association with cytochrome *c* [9], [11]. Previous proposals for the roles of subunits have relied exclusively on bioinformatic analyses and extrapolation from characterized NAD-dependent Rnf complexes of non-methanogenic species classified in the domain *Bacteria* [2], [11]. Here we report biochemical properties of RnfB and RnfG, key subunits that correspondingly interact with the electron donor and acceptor of the complex from *M. acetivorans*. The results advance an understanding of the organization and biochemical properties of the Rnf complex and lay a foundation for further understanding the overall mechanism in the pathway of methane formation from acetate.

## Results

### RnfG

**Figure 2:**
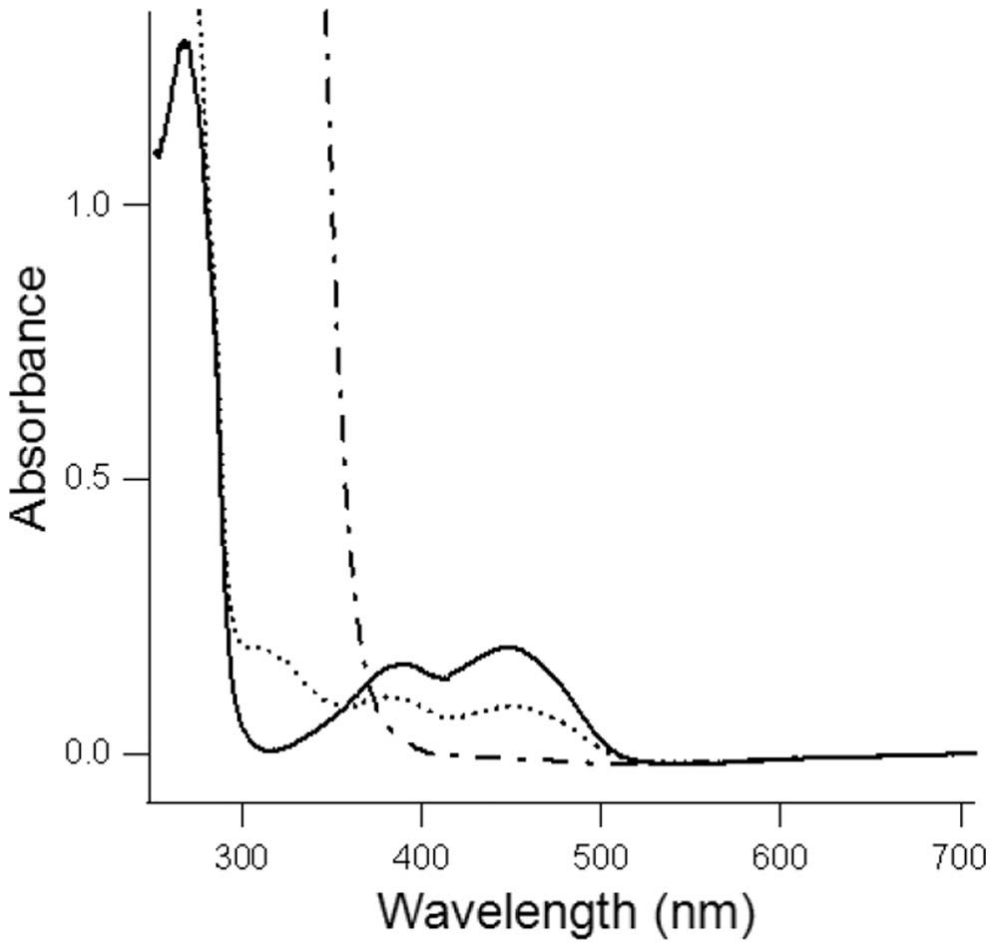
UV-visible spectrum of RnfG from *Methanosarcina acetivorans*. Symbols: fully oxidized (−), partially reduced with sodium dithionite (⋅⋅⋅⋅⋅), and fully reduced with sodium dithionite (−⋅−). The protein (25 µM) was contained in 25 mM Tris-HCl, pH 8.

The His-tagged RnfG subunit was overproduced in *E. coli* and purified to initiate investigations into electron transfer functions of this eight-subunit Rnf complex from the domain *Archaea*. The majority of the protein was present in the membrane fraction consistent with location in the membrane of *M. acetivorans*. SDS-PAGE (Fig. S1 in File S1) of the protein solubilized and purified from the membrane fraction indicated a molecular mass of 19+0.5 kDa corresponding to the theoretical value of 19.7 kDa. Western blot analysis showed the protein cross reacted with anti-His antibody confirming RnfG (not shown). UV illumination of the SDS-PAGE gel prior to protein staining revealed a corresponding fluorescent band consistent with a flavin cofactor (Fig. S1 in File S1). The UV-visible spectrum (Fig. 2) revealed maximum absorbance at 395 nm and shoulders at 456 nm and 475 nm typical of flavoproteins. The representative preparation shown in Fig. 2 contained a molar ratio of 0.84 flavin based on an ε_450_ of 12,200 M^−1^ cm^−1^ for free FMN [36]. Neither boiling nor treatment with TCA depleted the UV-visible absorbance or fluorescence. The results indicate that RnfG contains one covalently bound flavin.

Subunits of the Rnf complex from *M. acetivorans* share sequence identity with the NADH:quinone oxidoreductase (Nqr) respiratory complex that drives the transport of Na^+^ across the cell membrane of aerobic microbes generating an electrochemical gradient [37]. Sequence comparison of the RnfG subunit of *M. acetivorans* with the NqrC subunit from *Vibrio cholerae* (Fig. S2 in File S1) identified a common motif (S/TGATIT/S) with the conserved threonine shown to covalently bind FMN in NqrC. However, serine and histidine residues have also been discussed as a potential flavin binding site [24], [38]. Thus, Thr_166_ of the SGAT motif in RnfG from *M. acetivorans* was replaced with glycine by site-directed mutagenesis and the RnfG variant produced in *E. coli* to identify the covalent binding site for FMN. The purified variant was colorless and failed to fluoresce under UV illumination, a result indicating Thr_166_ is involved in the covalent binding of FMN in the RnfG subunit of *M. acetivorans*.

**Figure 3:**
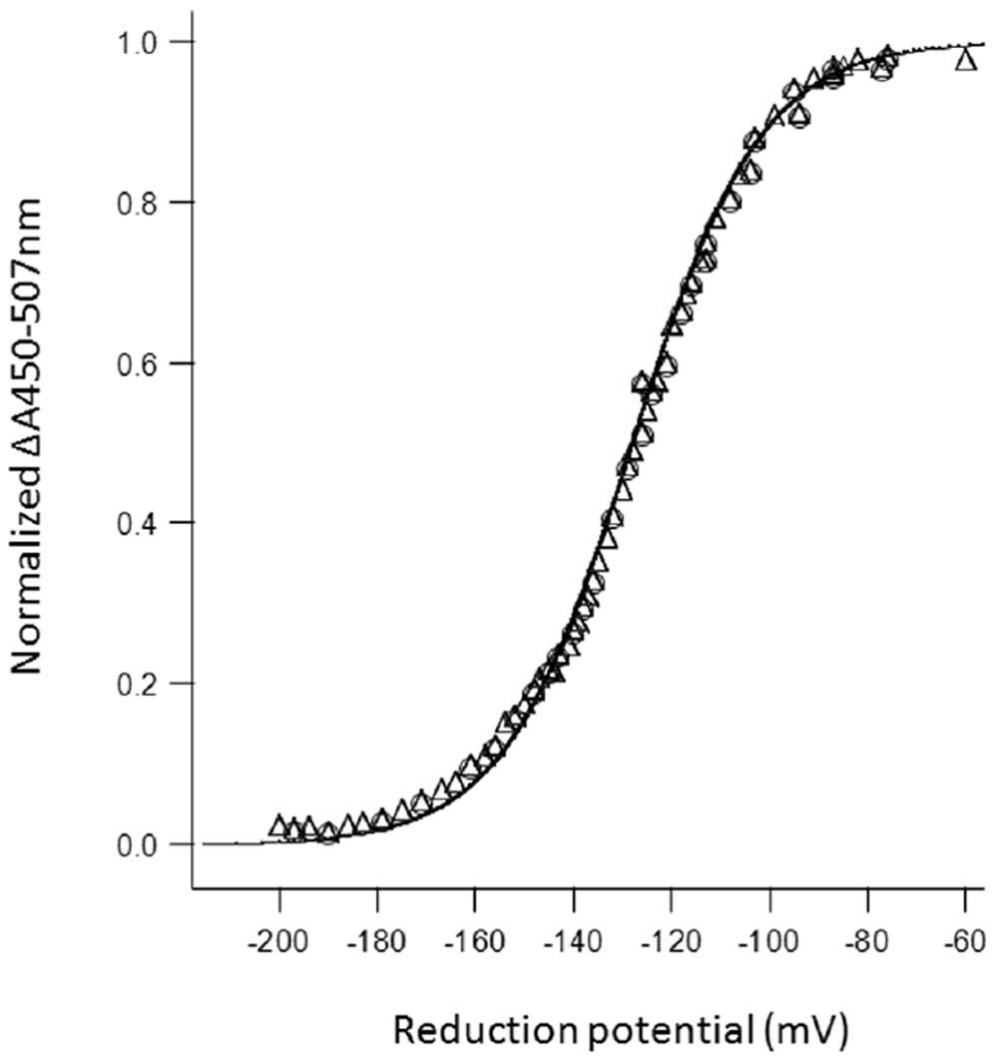
Redox titration of RnfG. Symbols: reduction (Δ), re-oxidation (o). The data were fit to the Nernst equation with *n* = 2.

Potentiometric titrations of FMN in RnfG at pH 7.5 revealed an *E*_m_ value of −129±5 mV with n = 2 (Fig. 3). The absence of absorbance between 500 and 700 nm for partially reduced RnfG suggests RnfG is less able to stabilize a neutral (red) semiquinone, and the spectrum of partially reduced RnfG did not reveal a shift of the 395 nm peak to lower wavelengths that would have indicated an anionic (blue) semiquinone (Fig. 2). Furthermore, a flavin radical was not observed by EPR spectroscopy in either the oxidized or reduced protein, and EPR signals were not observed in samples taken during equilibrium potentiometric titrations (not shown). These results indicate that the fully reduced hydroquinone is more thermodynamically stable than the semiquinone which is short-lived and undetectable.

**Figure 4:**
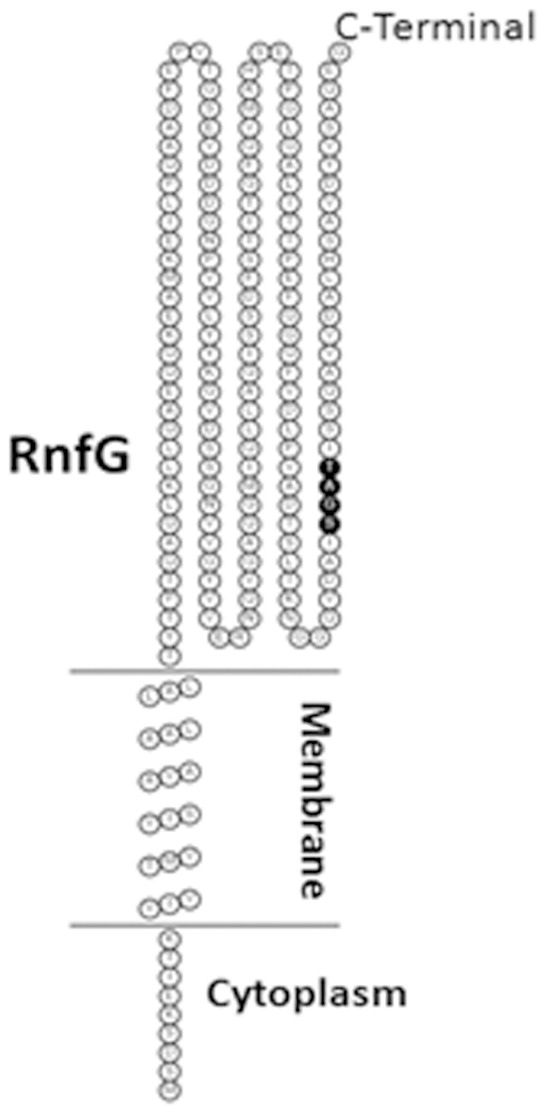
Membrane topology of RnfG predicted with the HMMTOP algorithm. The SGAT motifs is highlighted. The topology was displayed using TOPO2.

The finding that the heterologously-produced RnfG was primarily in the membrane fraction of *E. coli* suggests that the subunit is intrinsic to the membrane of *M. acetivorans*. Thus, the topology of the subunit was investigated to determine the location of the FMN cofactor necessary to provide a basis for understanding its function and interactions with other redox-active subunits of the Rnf complex. Both the TMHMM and MEMSAT3 topology prediction algorithms indicated that the secondary structure of RnfG contains a single helical transmembrane domain. The TMHMM algorithm predicted the transmembrane domain encompassing residues 7–29 (Fig. 4) and the MEMSAT3 algorithm predicted residues 7–30 (not shown) from the N-terminus. The predictions are in agreement with a cytoplasmic location of the N-terminus placing the C-terminus with the FMN binding site on the outer aspect of the membrane (Fig. 4).

**Figure 5:**
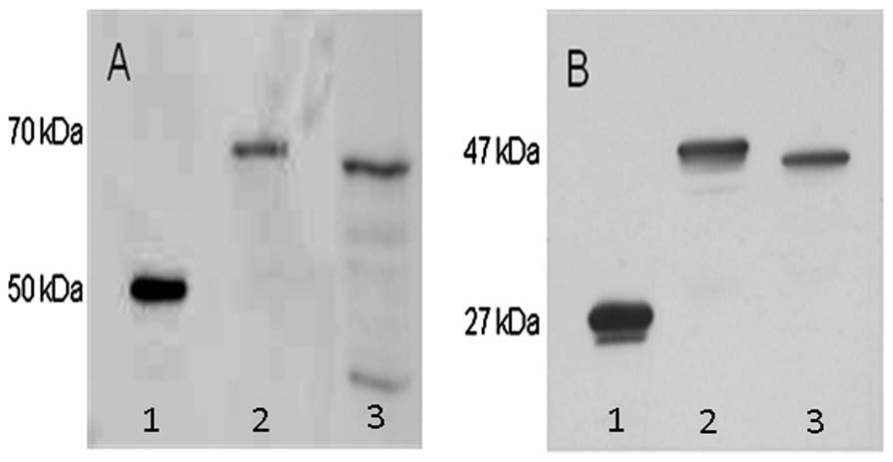
Western blots of RnfG fusion proteins. Panel A. Lane 1, alkaline phosphatase; lane 2, normal RnfG fused to alkaline phosphatase; lane 3, transmembrane-truncated normal RnfG fused to alkaline phosphatase. Panel B. Lane 1, GFP; lane 2, normal RnfG fused to GFP; lane 3, transmembrane-truncated RnfG fused to GFP.

**Figure 6:**
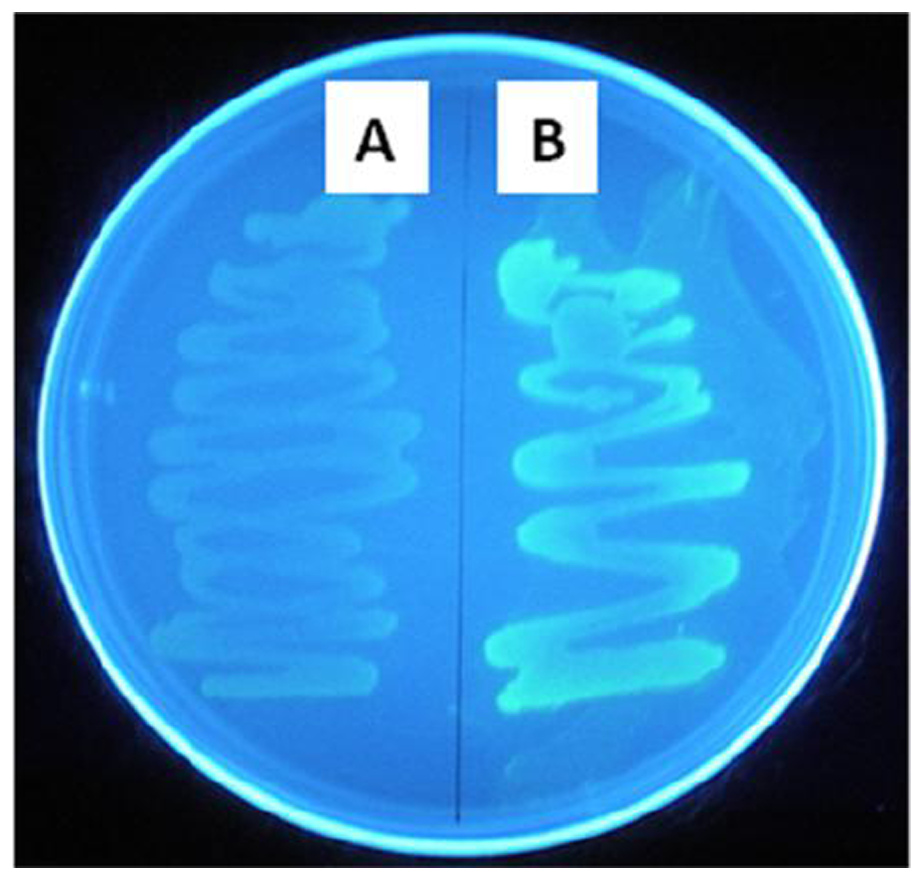
Fluorescence of *Escherichia coli* LMG 194 cells expressing RnfG fusion proteins. A side, normal RnfG fused to GFP. B side, transmembrane-truncated RnfG fused to GFP. The photo was taken under UV illumination.

In order to further investigate the location of the C-terminal domain and FMN binding site, *E. coli* TOPO 10 was transformed with genetic constructs encoding full-length and transmembrane-truncated RnfG fused at the C-terminus with alkaline phosphatase under control of the arabinose promoter. Heterologous production of the fusion proteins were confirmed by Western blotting using anti-alkaline phosphatase antibody (Fig. 5A). Alkaline phosphatase migrated with the predicted molecular mass of ∼50 kDa. The full-length RnfG fusion protein migrated with the predicted molecular mass of ∼70 kDa whereas truncated RnfG fusion protein migrated to the expected lower molecular mass. The alkaline phosphatase activity of permeabilized cells transformed with the full-length RnfG fusion construct was 570 U, while activity of cells transformed with the transmembrane-truncated RnfG fusion construct was 10 U. Since alkaline phosphatase is only active in the periplasm of *E. coli*, the results indicate that the location of the C-terminus adjacent to the FMN binding site is at the outer aspect of the cytoplasmic membrane in *M. acetivorans* consistent with that predicted by the topology algorithms. Similar experiments were also conducted with genetic constructs encoding full-sequence and transmembrane-truncated RnfG fused at the C-terminus with GFP. Expression of the fusion protein was confirmed by Western blotting using anti-GFP antibody (Fig. 5B). GFP migrated with the predicted molecular mass of approximately ∼27 kDa. The full-length RnfG fusion protein migrated with the predicted molecular mass of ∼47 kDa whereas the fused transmembrane-truncated RnfG migrated to the expected lower molecular mass. The fluorescence of cells transformed with the transmembrane-truncated RnfG fusion construct was markedly greater than that of cells transformed with the full-length RnfG fusion construct (Fig. 6). Since GFP is only fluorescent in the cytoplasm, the results are in agreement with the alkaline phosphatase fusion experiments indicating that the C-terminal domain of RnfG is located in the periplasm of *E. coli*. Overall, the results from the fusion experiments combined with topology modeling predictions indicate that the FMN binding site is located on the outer aspect of the *M. acetivorans* cytoplasmic membrane.

### RnfB

The His-tagged RnfB, produced primarily in the membrane fraction of *E. coli*, was purified to homogeneity as revealed by SDS-PAGE and identity confirmed by Western blotting with anti-His tag antibody (Fig. S3 in File S1). The band migrated with an apparent molecular mass of 27±0.5 kDa close to the theoretical 30.5 kDa. A minor band was detected after affinity chromatography. The intensity ratio of the bands was unchanged during protein purification, freezing and thawing cycles or by varying the SDS concentration suggesting the 27 kDa band is not due to protein degradation. The secondary structure prediction (Fig. S4 in File S1) revealed an N-terminal transmembrane helix consistent with the membrane location when produced in *E. coli*, results suggesting RnfB is anchored to the membrane of *M. acetivorans*. This conclusion is in agreement with that reported for RnfB of *R. capsulatus* that also contains an N-terminal transmembrane domain [39]. The discrepancy between the calculated (30 kDa) and observed (27 kDa) molecular mass (Fig. S3 in File S1) is most probably caused by hydrophobicity of the transmembrane domain based on the finding that hydrophobic proteins migrate faster than hydrophilic proteins, the result of only partial unfolding in the presence of SDS [40], [41].

**Figure 7:**
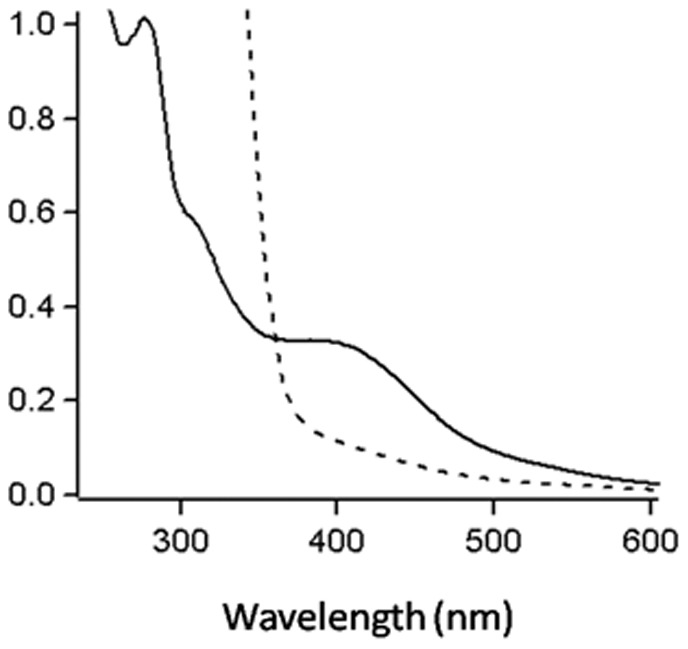
UV-visible spectra of the RnfB from *Methanosarcina acetivorans*. Oxidized (−), fully reduced with sodium dithionite (⋅⋅⋅⋅⋅).

The deduced sequence of the gene encoding RnfB (MA0664) revealed three CX_2_CX_2_CX_3_CP motifs typical of ligating 4Fe-4S clusters (Fig. S4 in File S1). The as-purified protein was dark brown with a UV-visible spectrum typical of oxidized iron-sulfur proteins showing a major peak centered at 400 nm and a shoulder centered at 320 nm (Fig. 7). The 400 nm peak disappeared upon reduction with sodium dithionite. The average moles of iron and acid-labile sulfide per mole of protein were found to be 6.5±0.4 and 7.3±0.5 (n = 3) respectively. These results confirm that RnfB from *M. acetivorans* is an iron-sulfur protein. Attempts to reconstitute the protein with iron and sulfide following described methods [42] did not alter the 280/400 nm absorbance ratio or the content of iron and acid-labile sulfide.

**Figure 8:**
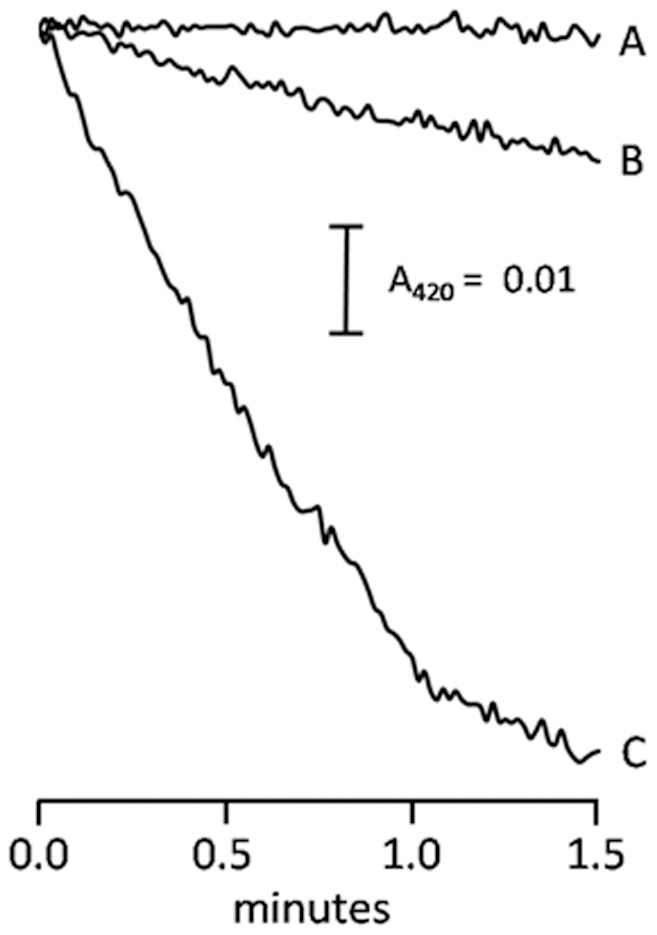
Ferredoxin dependent reduction of RnfB by CdhAE. The complete reaction mixture contained 34 µM RnfB, 1.3 µM ferredoxin and 180 µM CdhAE in 50 mM MOPS buffer (pH 6.8) under 1 Atm CO. The reaction was initiated by addition of CdhAE pre-reduced under 1 Atm CO. Trace A, complete reaction mixture minus RnfB. Trace B, complete reaction mixture minus ferredoxin. Trace C, complete reaction mixture.

It has been hypothesized that RnfB is the entry point of electrons to Rnf complexes from the domain *Bacteria* that generate ion gradients and that Fd is the electron donor [43], [44]. Ferredoxin was previously shown to accept electrons from the CdhAE component of the Cdh complex of *M. acetivorans* and donate electrons to the membrane-bound electron transport chain [9], although the entry point was not identified. Thus, the ability of RnfB to be reduced by Fd was evaluated in a system comprised of purified CdhAE, Fd and RnfB. The Fd used in the assay was purified from acetate-grown *M. acetivorans* as previously described [9]. CO was the electron donor to CdhAE, a surrogate for the carbonyl group of acetyl-CoA. Figure 8 shows that the Fd mediates electron transfer from CdhAE to RnfB. Importantly, the results indicate that no other electron carriers are necessary to mediate electron transfer from the Cdh complex to the Rnf complex which establishes the initial steps of the electron transport pathway operable during acetotrophic growth.

**Figure 9:**
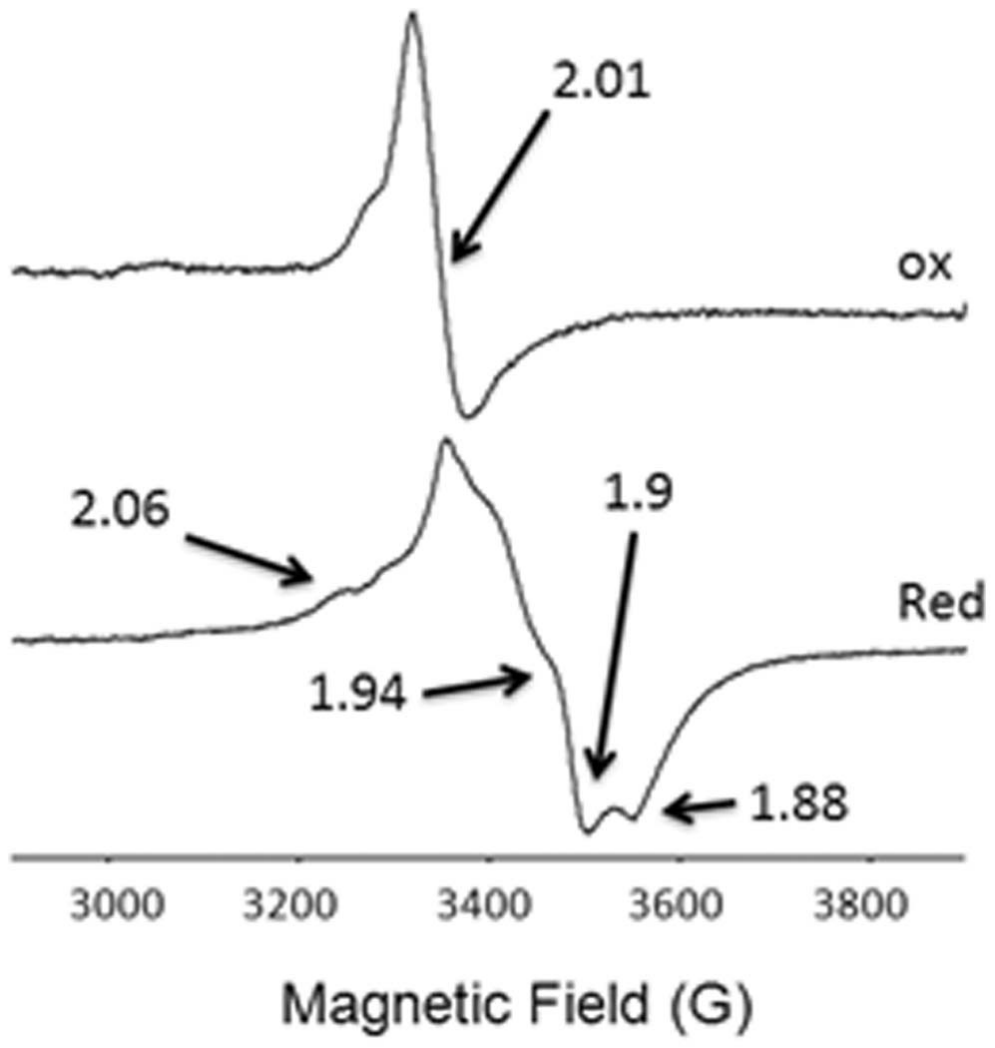
X-band EPR spectra of oxidized (upper trace) and dithionite-reduced (lower trace) of RnfB from *Methanosarcina acetivorans*. The characteristic lines for the 7Fe-8S center are indicated by the *g*-values. EPR conditions: Frequency: 9.41 GHz; microwave power: 2 mW; modulation amplitude: 1 mT; temperature: 12 K.

Rnf B was further investigated by EPR spectroscopy. The spectrum of the oxidized enzyme showed a signal around *g* = 2.01 (Fig. 9, upper trace) characteristic of a 3Fe-4S center (49–51). However, uncharacteristic of 3Fe-4S centers, this signal disappeared upon reduction by dithionite (Fig. 9, lower trace) and was replaced by a broad signal with *g* values of 2.06, 1.94, 1.90, 1.88. This spectrum is atypical of magnetically isolated reduced 4Fe-4S clusters that generally show a maximum of three well resolved lines. Instead, the spectrum suggests a dipolar magnetic interaction between the reduced 3Fe-4S cluster (S = 2) and a 4Fe-4S cluster (S = ½) similar to that reported for 7Fe Fds containing magnetically coupled 3Fe-4S and 4Fe-4S clusters (52,53). The spectroscopic results combined with the iron-sulfur content and deduced sequence shows that RnfB contains multiple iron-sulfur clusters suggesting it functions as a “wire conduit” coupling Fd with redox-subunits of the Rnf complex.

## Discussion

Rnf complexes are widely distributed in species with diverse metabolisms from the domains *Bacteria* and *Archaea*. Remarkably, biochemical characterizations are reported for only two Rnf complexes that function in electron transport pathways distinct from the pathway for conversion of acetate to methane [24], [35]. The results presented are the first for an Rnf complex from a methane-producing species and the first from the domain *Archaea*. The results establish that the RnfG subunit of the *M. acetivorans* Rnf complex contains covalently attached FMN positioned on the outer aspect of the cytoplasmic membrane with a redox potential of -129 mV. The RnfG subunit from *M. acetivorans* of the domain *Archaea* both compares and contrasts with the characterized RnfG subunit of the Rnf complex from *V. cholerae* of the domain *Bacteria* [24]. Both subunits bind FMN covalently to the conserved threonine residue in the S/TGAT motif. This finding in organisms with diverse metabolisms from the domains *Archaea* and *Bacteria* underscores the universality and importance of the motif in binding flavin. Indeed, the motif also binds FMN covalently to subunits of the Na^+^-pumping NADH:quinone oxidoreductase (Nqr) in species from the domain *Bacteria* [45]. On the other hand, RnfG from *V. cholerae* appears to stabilize the semiquinone of FMN more readily than the RnfG from *M. acetivorans* suggesting different functions that potentially reflect differences in electron acceptors for these complexes.

Characterization of the heterologously-expressed RnfB identified the presence of multiple iron-sulfur clusters and that it accepts electrons from the Fd that functions during growth of *M. acetivorans* with acetate. The evidence for multiple iron-sulfur clusters suggest RnfB functions as a “wire conduit” coupling electron transfer from Fd to the redox-active subunits of the Rnf complex. The evidence presented for 3Fe-4S and 4Fe-4S clusters in RnfB is consistent with the iron and acid-labile sulfur content. However, analysis of the deduced sequence identified three CX_2_CX_2_CX_3_CP motifs capable of ligating iron-sulfur clusters. Furthermore, a CX_2_CX_4_CX_16_CP motif present in *M. acetivorans* RnfB is also present in the CdhE and AcsC subunits of the Cdh complex of *Methanosarcina thermophila* and the acetogen *Moorella thermoacetica* (Fig. S4 in File S1) shown to ligate a 4Fe-4S cluster [46]–[49]. Thus, although attempts to reconstitute additional iron-sulfur in the *M. acetivorans* RnfB were unsuccessful, it cannot be ruled out that additional clusters are present in the protein synthesized by *M. acetivorans*. In contrast to *M. acetivorans*, the *R. capsulatus* RnfB contains two CX_2_CX_2_CX_3_CP motifs and a motif similar to CX_2_CX_4_CX_16_CP of *M. acetivorans* (Fig. S4 in File S1) although the iron and acid-labile sulfur content, UV-visible spectra and EPR spectra indicate a single 2Fe-2S [39]. The differences potentially signal differences in the proposed functions of the Rnf complexes from *M. acetivorans versus R. capsulatus*. Ferredoxin is the postulated electron acceptor for RnfB in the *R. capsulatus* Rnf complex that oxidizes NADH and reduces Fd in a reversed electron transport [35], [50], [51].

**Figure 10:**
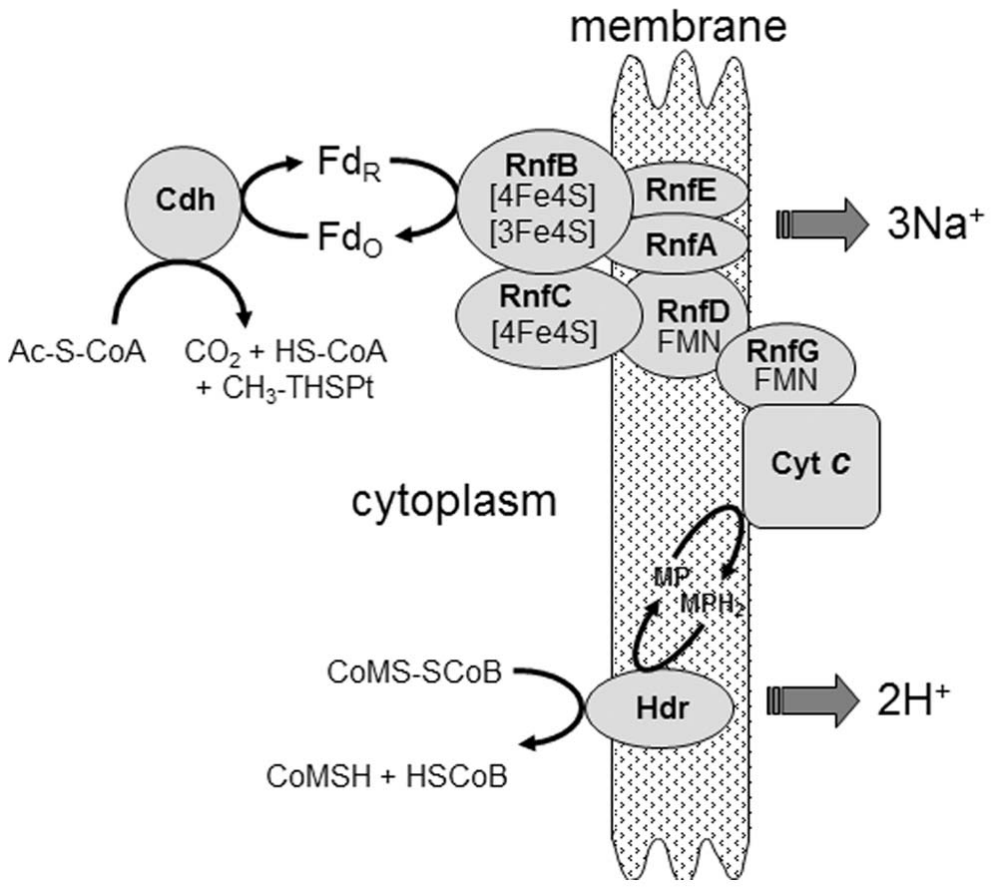
Proposed organization of the Rnf complex from *Methanosarcina acetivorans*. Symbols: Fd_O_, oxidized ferredoxin; Fd_R_, reduced ferredoxin.

The results presented lead to a revised and testable model (Fig. 10) for the organization and function of subunits in the Rnf complex of *M. acetivorans*. In the model, RnfB functions as an electron conducting wire transferring electrons from Fd to RnfC. RnfC has no transmembrane-spanning sequences consistent with a cytoplasmic-facing membrane component as proposed for other Rnf complexes [37], [43] for which the RnfC subunit interacts with NADH. However, the Rnf complex from *M. acetivorans* does not interact with NAD or NADP requiring an alternate function. It was previously reported that the sequence of RnfC from *M. acetivorans* contains two motifs consistent with ligation of two 4Fe-4S clusters [26] indicating an electron transport function. Topology analyses of RnfD indicate several transmembrane spanning regions and sequence analyses indicate a potential flavin binding motif SGTF (Fig. S5 in File S1). Thus, RnfC and RnfD are proposed to reside in an electron transfer sub-complex with RnfB. The results presented support RnfG, with the flavin cofactor exposed to the periplasmic side of the membrane, as the distal electron acceptor in the proposed sub-complex (Fig. 10). RnfB was unable to reduce RnfG consistent with the proposed model. Topology analyses indicated that the RnfA, RnfD and RnfE subunits from *M. acetivorans* contain six hydrophobic transmembrane spanning helices suggesting these subunits are integral to the membrane (Fig. S5 in File S1). The deduced sequences of RnfA and RnfE show no recognizable motifs ligating redox cofactors which excludes an electron transport function. Thus, it is proposed that RnfA and RnfE cooperate with the electron transport sub-complex to translocate Na^+^ outside the membrane driven by electron transport from RnfB to RnfG. Sodium was previously reported to be translocated by the intact Rnf complex of *M. acetivorans* [14]. Candidates for the electron acceptor of RnfG are MP and the multi-heme cytochrome *c* shown previously to participate in the Fd:CoMS-SCoB oxidoreductase system [9]. The standard redox potential of -165 mV determined for MP [52] would appear to preclude it as the electron acceptor for RnfG (*E*_m_ = −129 mV); however, the redox potential for RnfG may be more negative when in the membrane environment and in association with other subunits of the Rnf complex. The location of cytochrome *c* is predicted (Fig. 10) to reside outside the cytoplasmic membrane based on precedent for the location of multi-heme cytochromes *c* in prokaryotes [53]. The proposed location of the RnfG flavin binding site outside the membrane infers a potential interaction between RnfG and cytochrome *c*. Thus, the model in Figure 10 postulates electron transfer from RnfG to cytochrome *c* that donates electrons to MP which is the proposed electron donor to the heterodisulfide reductase HdrDE [2], [9]. We postulate that *M. acetivorans* adapted the six-subunit Rnf complex from non-methanogenic species of the domain *Bacteria* by incorporating cytochrome *c* into the complex to interface with the MP-dependent HdrDE. Co-transcription of the gene encoding cytochrome *c* with genes encoding all other Rnf subunits is consistent with this hypothesis.The proposed roles for RnfG and RnfC (Fig. 10) contrast with functions proposed for homologs in the majority of Rnf complexes from species in the domain *Bacteria* [37] that are NAD dependent. In *M. acetivorans*, RnfC is postulated to mediate electron transfer from Fd to RnfG whereas the bacterial homolog of RnfC is proposed to interact with NAD. The results presented here suggest RnfG in *M. acetivorans* is the terminus of an electron transfer sub-complex donating electrons to cytochrome *c* whereas the bacterial homolog of RnfG mediates electron transfer to RnfC that interacts with NAD. These differences reflect the different physiological functions of NAD-dependent Rnf complexes from species in the domain *Bacteria versus* the complex from *M. acetivorans* classified in the domain *Archaea*.

### Conclusions

Although Rnf complexes are widely distributed in diverse microbes, biochemical characterizations are only reported for selected subunits from two species in the domain *Bacteria*. Key subunits from the Rnf complex of *M. acetivorans*, interacting with the electron donor and acceptor, have been characterized. The results advance an understanding of the organization and predicted function of subunits in the complex and provide direction for further research. Finally, the results contrast Rnf complexes from physiologically diverse species in the domains *Bacteria* and *Archaea* that contributes to a broader understanding of the expanding family of Rnf complexes.

## Materials and Methods

### Heterologous expression of RnfB, RnfG and the RnfG T166G variant

Strains and plasmids used in this study are shown in Table 1. The ORFs (MA0664 and MA0661) encoding RnfB and RnfG were amplified by PCR from genomic DNA of *M. acetivorans* and cloned into a pBAD TOPO T/A vector (Invitrogen). A pair of primers, sense (^5′^GAGGAA*TAATAA* ATGAGTAGTGTGCTCATAAACTC) and antisense (^5′^ CCGGAGCTCGATTGCCTTC), was used to amplify the *rnfB* gene. The sense (^5′^GAGGAA*TAATAA*ATGAGTGATAGTAAGGAAATAAC) and antisense (^5′^ GCCCTCCTGTGCGGATACATAATCAACTGC) primers were used to PCR amplify the *rnfG* gene. In order to remove the N-terminal leader in the vectors, stop codons (shown italicized) and a translation re-initiation sequence consisting of a ribosomal binding site (shown underlined) were introduced in the sense primer before the ATG start codon (shown underlined). The antisense primer was designed to exclude the stop codon during amplification in order to extend the protein product at the C terminus with a six-histidine tag encoded in the pBAD TOPO T/A cloning vector. The PCR reaction was done using the Fast Start High Fidelity PCR system (Roche) which generates T/A overhangs. The PCR products were cloned into the pBAD TOPO T/A vector yielding the pBAD-RnfB and pBAD-RnfG plasmids. The recombinant plasmids were used to transform One Shot TOP10 *E. coli* (Invitrogen). The recombinant plasmids containing the DNA insert were confirmed by completely sequencing each plasmid that was transformed into *E. coli* LMG194 (Invitrogen). The transformed strains were cultured at 37°C in RM medium containing 100 mg/l of ampicillin. Production of RnfB and RnfG was induced by the addition of 0.2% (w/v) arabinose per liter of culture when the OD_600_ reached 0.7. The induced cultures were then incubated for another 16 h at 27°C. Cells were harvested by centrifugation and stored at −80°C. The production of RnfB and RnfG was confirmed by Western blotting of crude cell extract using anti-His antiserum coupled to alkaline phosphatase (Invitrogen).

**Table 1:** Strains and plasmids use in this study.

Strain or plasmid	Genotype or description	Source or reference
**Strains**		
*E. coli* TOP10	F^-^ *mcr*A Δ(*mrr*-*hsdRMS*-*mcrBC*) φ80*lacZ*Δ*M15* Δ*lacX74 recA1 araD139*Δ (*ara-leu*)*7697 galU galK rpsL* (Str^r^) *endA1 nupG*	Life Technologies
*E. coli* LMG 194	F*-*Δ*lacX74 galE thi rpsL* Δ*phoA*(PvuII) Δ*ara714 leu*::Tn10	Life Technologies
**Plasmids**		
pBAD-GFP	pBAD containing cycle 3 *gfp*	[58]
pBAD-PhoA	pBAD22 derivative containing signal sequenceless *phoA* with a 5′ KpnI site	[57]
pBAD-RnfB	pBAD encoding full-length RnfB	This study
pBAD-RnfG	pBAD encoding full-length RnfG	This study
pBAD-RnfGT166G	pBAD encoding the full-length RnfG T166G variant	This study
pBAD-phoAFLG	pBAD-phoA encoding PhoA C-terminal fusion to full-length RnfG	This study
pBAD-phoATRG	pBAD-phoA encoding PhoA C-terminal fusion to truncated RnfG	This study
pBAD-GFPFLG	pBAD-GFP encoding GFP C-terminal fusion to full-length RnfG	This study
pBAD-GFPTRG	pBAD-GFP encoding GFP C-terminal fusion to truncated RnfG	This study

The variant RnfG-T166G protein was derived by site directed mutagenesis of pBAD-RnfG, replacing the threonine codon (ACG) with the glycine codon (GGC) using the sense primer (5′GCCATTTCCGGAGCTGGCATCTCTTCACAGGCAGTG) and the antisense primer (5′ CACTGCCTGTGAAGAGATGCCACGTCCGGAAATGGC. The mutant pBAD-RnfG-T166G was also introduced in *E. coli* LMG194 and the variant protein overproduced as described below for normal RnfG.

### Purification of RnfG

The predicted secondary structure suggests that RnfG is a membrane bound protein with one N-terminal α-helix. In order to confirm this prediction, RnfG was initially purified aerobically from both membrane and soluble fractions of *E. coli*. A greater amount was purified from the membrane fraction and, therefore, RnfG was routinely purified aerobically from *E. coli* membranes. The purification of RnfG was visibly monitored due to the distinct yellow color of flavin. Purification of the colorless variant was achieved by following the precise procedure determined for normal RnfG. About 100 g wet weight of cells were thawed and re-suspended in 20 mM potassium phosphate buffer (pH 7.4) containing 500 mM NaCl, DNAse and 0.25 mM phenylmethylsulfonyl fluoride. The cells were lysed by passing twice through a French pressure cell at 110 MPa. The membrane was separated from the soluble fraction by high speed centrifugation at 110,000×*g* for 60 min at 4°C. The supernatant was discharged and the pellet of membranes were re-suspended to a final concentration of 10 mg/ml protein in 20 mM potassium phosphate buffer (pH 7.4) containing 500 mM NaCl, 20 mM imidazole, DNAse and 0.25 mM phenylmethylsulfonyl fluoride. Proteins were solubilized from the membrane suspension by adding a 10% n-dodecyl-β-D-maltoside solution drop wise to a final concentration of 1% followed by incubation at 4°C for 20 min with slow agitation. Lipid was removed by centrifugation at 65,000×*g* for 45 min at 4°C. The supernatant solution was loaded onto a Ni Sepharose Fast flow column (Amersham Biosciences) equilibrated with 20 mM phosphate (pH 7.4) containing 500 mM NaCl, 20 mM imidazole, and 0.05% dodecyl-β-D-maltoside. The column was then washed with 3 column volumes of 40 mM phosphate (pH 7.4) containing 500 mM NaCl, 50 mM imidazole, 0.01% dodecyl-β-D-maltoside and 25% glycerol. After washing with another column volume of washing buffer without glycerol, RnfG or RnfG-T166G were eluted with 20 mM phosphate (pH 7.4) containing 500 mM NaCl and 250 mM imidazole. The fractions containing the normal or variant proteins were concentrated using a Centricon YM-10,000 membrane (Millipore). The concentrated fraction was then diluted 20-fold with 20 mM HEPES buffer (pH 7.8) containing 0.01% dodecyl-β-D-maltoside. The protein was then loaded onto a HiTrap Q-sepharose column equilibrated with 20 mM Tris-HCl buffer (pH 8.5) containing 0.01% dodecyl-β-D-maltoside. The column was washed with 20 mM Tris-HCl buffer (pH 8.5) containing 150 mM NaCl and 0.01% dodecyl-β-D-maltoside. The fraction containing RnfG or RnfG-T166G was eluted with 20 mM Tris-HCl (pH 8.5) containing 200 mM NaCl and 0.01% dodecyl-β-D-maltoside. This fraction was then diluted 10-fold with 20 mM Bis-Tris buffer (pH 6) containing 0.01% dodecyl-β-D-maltoside and subsequently washed with 20 mM Bis-Tris (pH 6) containing 150 mM NaCl and 0.01% dodecyl-β-D-maltoside. Finally, the purified proteins were eluted with 20 mM Bis-Tris buffer (pH 6) containing 200 mM NaCl and 0.01% dodecyl-β-D-maltoside.

### Redox titration of RnfG

Anaerobic dye-mediated redox titrations were carried out in a stoppered quartz cuvette as described previously [54] at 22°C. Into a solution of 30 *µ*M RnfG in 50 mM Tris-HCl (pH 7.5) were added the following redox mediators each at a concentration of 1 *µ*M: phenazine methosulfate, phenazine ethosulfate resorufin, indigocarmin, 2-hydroxy-1,4-naphthoquinone, anthraquinone 2-sulfonate, and benzyl viologen. The solution was kept anaerobic by continuously flushing with argon 6.0. Reductive titrations were performed by adding small aliquots from a freshly prepared anaerobic sodium dithionite solution and oxidative titrations by adding anaerobic potassium ferricyanide. UV-visible spectra were recorded with a HP8253 photodiode array spectrometer. Both a platinum electrode and an Ag/AgCl electrode were used to measure the redox potential. The electrodes were calibrated with a saturated solution of quinhydrone at pH 4.0 and 7.0. The quoted redox potentials are versus the normal hydrogen electrode (NHE).

### Localization of RnfG

The topology prediction was carried out using TMHMM [55] and MEMSAT3 [56] algorithms. Expression vectors encoding C-terminal fusions of alkaline phosphatase and green fluorescence protein (GFP) to full-length and transmembrane-truncated RnfG were constructed by insertion of the RnfG genes into *p*BAD-*gfp* [57] and pBAD-*pho*A [58] at *Nhe*I and *Kpn*I sites, respectively. The gene encoding truncated RnfG was constructed by deletion of nucleotides encoding the first 27 N-terminal residues which include the 18 transmembrane residues. The constructs were used to transform *E. coli* TOPO 10. The *E. coli* cells carrying the GFP gene were cultured on LB agar containing 0.2% arabinose. Fluorescent colonies were was identified by UV illumination. The *E. coli* cells carrying the alkaline phosphatase gene were cultured in liquid LB media at 37°C. Arabinose was added to a final concentration of 0.2% at an OD_600_ of 0.6 and the cells continuously grown for 4 h at 30°C. The alkaline phosphatase activity of permeabilized cells was determined as previously described [59]. The expression of fusion proteins were also analyzed using Western blotting with the corresponding antibody. The *E. coli* cells were grown in liquid LB media and the expression of fusion proteins were induced by addition 0.2% of arabinose at OD_600_ = 0.6. Cells were collected after 1 hour of additional incubation time and about 3 µg total protein was loaded onto SDS-PAGE gels. The expressed fusion proteins carrying GFP was detected using mouse anti-GFP monoclonal primary antibody (Millipore) and HRP anti-mouse secondary antibody. The expressed fusion proteins carrying alkaline phosphatase were detected using mouse anti-*E. coli* alkaline phosphatase monoclonal primary antibody (Millipore) and HRP anti-mouse secondary antibody. All exposure times were 1 min except for the transmembrane truncated RnfG fused with alkaline phosphatase that was exposed for 15 min.

### Purification of RnfB

The purification was performed in an anaerobic chamber (Coy Manufacturing) containing a 95% N_2_ and 5% H_2_ atmosphere. About 100 g wet weight of thawed cells were suspended in buffer A (20 mM potassium phosphate, pH 7.4) containing DNAse and 0.25 mM phenylmethylsulfonyl fluoride. The suspension was passed twice through a French pressure cell at 110 MPa and the lysate centrifuged at 110,000×*g* for 60 min at 4°C. The supernatant solution containing the soluble protein fraction was removed and the pellet containing the membrane fraction re-suspended to a final concentration of 10 mg protein/ml in buffer A containing 500 mM NaCl, 20 mM imidazole, DNAse and 0.25 mM phenylmethylsulfonyl fluoride. Membrane proteins were solubilized by drop-wise addition of a 10% (w/v) n-dodecyl-β-D-maltoside solution to a final concentration of 1%, and incubated at 4°C for 20 min with slow agitation. Lipid was removed by centrifugation at 65,000×*g* for 45 min at 4°C. Western blotting of the soluble and solubilized membrane fraction revealed the great majority of RnfB in the membrane fraction from which RnfB was purified. The supernatant solution obtained by solubilization of the membrane fraction was loaded onto a Ni Sepharose Fast Flow (column (1.5×15 cm) (GE Healthcare) equilibrated with buffer A containing 500 mM NaCl, 20 mM imidazole, and 0.05% (w/v) dodecyl-β-D-maltoside. The column was then washed with 3 column volumes of buffer B (40 mM potassium phosphate, pH 7.4) containing 500 mM NaCl, 50 mM imidazole and 0.01% (w/v) dodecyl-β-D-maltoside. Proteins were eluted with Buffer A containing 500 mM NaCl and 150 mM imidazole. The eluted brown fraction was concentrated to 5 ml using a Vivacell fitted with a 70 Polythersulfone 10,000 MWCO membrane filter (Sartorius Stedim). The concentrate was diluted 20-fold with buffer C (20 mM Tris-HCl, pH 8.5) containing 0.01% (w/v) dodecyl-β-D-maltoside and loaded onto a HiTrap Q-sepharose Fast Flow column (1.6×2.5 cm) (GE Healthcare) equilibrated with the dilution buffer. The column was washed with buffer C containing 250 mM NaCl and 0.01% (w/v) dodecyl-β-D-maltoside. The column was developed by batch elution with 15 ml of buffer C containing 500 mM NaCl and 0.01% (w/v) dodecyl-β-D-maltoside. The brown fraction was diluted 10-fold with buffer D (20 mM Bis-Tris, pH 6) containing 0.01% (w/v) dodecyl-β-D-maltoside and loaded onto a HiTrap SP Fast Flow column (1.6×2.5 cm) (GE Healthcare). The flow-through fraction containing RnfB was collected and used for further analysis.

### Purification of CdhAE and ferredoxin

Purification of the CdhAE component of the Cdh complex and Fd from acetate-grown *M. acetivorans* was as previously described [9].

### Analytical

Iron and acid labile sulfur content was determined as previously described [60], [61]. Each determination was the average of three replicates. The X-band EPR data were recorded using a Bruker ER200D EPR spectrometer with a home built He-flow system [62]. The microwave frequency was measured with an HP5350B frequency counter.

## Supporting Information

File S1 **Supporting figures. Figure S1, SDS-PAGE of purified normal RnfG and variant RnfG-T166A from** ***Methanosarcina acetivorans*** **. Figure S2, Sequence alignment of RnfG from** ***Methanosarcina acetivorans*** **with NqrC from** ***Vibrio cholerae*** **. Figure S3, SDS-PAGE of RnfB from** ***Methanosarcina acetivorans*** **. Figure S4, Sequence comparisons of the RnfB subunit from** ***Methanosarcina acetivorans*** **. Figure S5, Membrane topology of RnfA, RnfD and RnfE predicted with the HMMTOP algorithm.**
(PDF)
