# Supplementary material for: Characterization of the RnfB and RnfG Subunits of the Rnf Complex from the Archaeon Methanosarcina acetivorans
Source: PLoS One. 2014 May 16;9(5):e97966. doi: 10.1371/journal.pone.0097966 (PMC4023990; doi:10.1371/journal.pone.0097966)

Supporting Information

Figure S1. SDS-PAGE of purified wild-type RnfG and variant RnfG-T166A from *Methanosarcina acetivorans*. Lane 1, molecular mass standards; lane 2, purified RnfG (15 µg); lane 3, purified RnfG-T166A (15 µg). Lanes 1-3 were stained with Coomassie brilliant blue R250. Lanes 4 and 5 correspond to lanes 2 and 3 exposed to UV illumination prior to protein staining.

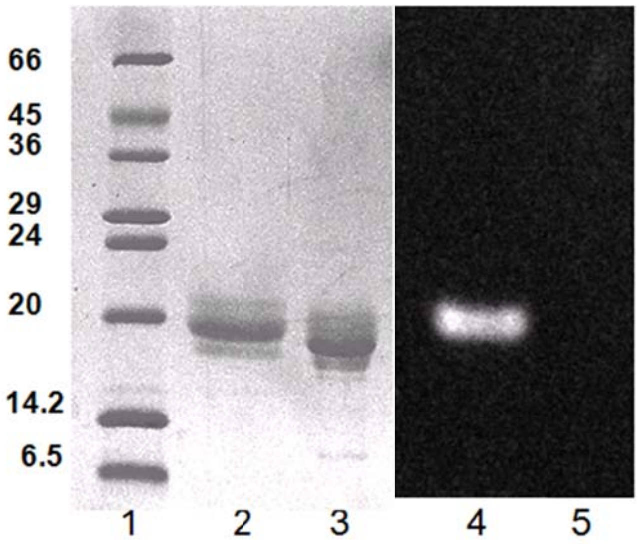

Figure S2. Sequence alignment of RnfG from *Methanosarcina acetivorans* with NqrC from *Vibrio cholerae*. Only the C-terminal domains are aligned showing potential FMN-binding motifs (**bold and underlined**) previously proposed for NqrC (). The threonine 166 residue of *M. acetivorans* RnfG is shown in ***bold italic***. Shaded residues are potential FMN-binding sites for RnfG. Aligned with NCBI blasp. RnfG, MA0661, gene id 1472553; NqrC, VC2293, gene id 4808844.

```
NqrC GLWSMMYAFVAVETDGNTVSGLTYYEQGETPGLGGEVENPAWRAQWVGKKLFDENHKPAI 203
     G   M+     + +D +T++G    +  ETPGLG  +  P ++ Q+V   + D +
RnfG GAQGMIQLLAGISSDFSTITGFQVMKHSETPGLGALITTPEFQGQFVDLPVADTS----- 151

NqrC KIVKGGAPQGSEHGVDGLSGATLTSNGV 231
      + K G        VD +SGAT++S  V
RnfG -LTKNGGQ------VDAISGATISSQAV 172
```

Figure S3. SDS-PAGE of RnfB from *Methanosarcina acetivorans*. Lane 1, molecular weight standards; Lane 2, purified RnfB stained with Coomassie blue R250; Lane 3, Western blot of purified RnfB using anti-His antibody coupled to alkaline phosphatase. The lanes were loaded with 15 µg of protein.

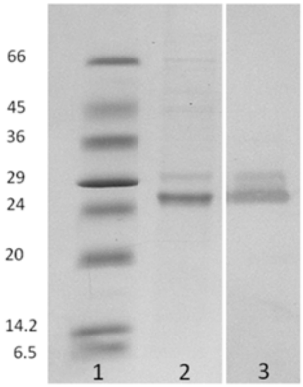

Figure S4. Sequence comparisons of the RnfB subunit from *Methanosarcina acetivorans*. Panel A, alignment with RnfB from *Rhodobacter capsulatus.* The predicted N-terminal transmembrane helix using the TMHMM and HMMTOP algorithms [54] is highlighted in grey and conserved cysteines are highlighted in black. Panel B, alignment with partial sequences of CdhE and AcsC subunits from the CO dehydrogenase/acetyl-CoA synthase complex of *Methanosarcina thermophila* (*M.t.*), *Moorella thermoacetica* (*M.th.*), and RnfB from *Rhodobacter capsulatus* (*R.c.*).

```
    (A)
M.a. MSSVLINSIAVLAGLGFAVGVMLVIASKVFKIDSNPLIDDVASLLPGANCGGCGFAGCAACAEAIVEQGAPVNSC 75
R.c. ----MIAAAASMSALGLGLGYLLGAAARKFHVETPPIVEEIAKILPGTNCGACGFPGCNGLAEAMAEGNAPVTAC 71
          *   *    **   *  *  *   *     *     *  *** *** *** **   ***  *  ***  *

M.a. PVGGFEVAKQIGALLGQEVTESEKEFPFVRCQGGNQHCTTLYDYHGVENCKVALMLCDSRKGCTYGCLGLGTCVQ 150
R.c. TPGGRDVALALAEIVTVEAG------------------------------------------------------- 91
       **  **         *

M.a  ACQFGALSMGEDGFPVVNKALCTSCGNCIAACPNGVLTFARDSEKVHVLCRSHDKGKDVKAVCEVGCIGCKKCEK 225
R.c. -ADAGPIAEIEPMVAFVFEDHCTGCQKCFKRCPTDAIVGG---------------AKQIHTVVMDACIGCDACIE 150
         *     *     *    ** *  *   **                       *    *    ****  *

M.a. ECPAGAIRVTEFLAEIDQEKCTACGACVAICPQKAIELR 264
R.c. VCPTEAIVSRVKPKTLKTWYWDKPQPGLVAASAETAA   187
      **

    (B)
M.a.  43 SLLPGANCGGCGFAGCAACAEAIVEQGAPVNSCPVGGFEV 82
R.c.  39 KILPGTNCGACGFPGCNGLAEAMAEGNAPVTACTPGGRDV 78
M.t.  11 KYLPQTNCGECGEPTCMAFASKLIDRSGKTSDCPPLVKEK 50
M.th.  9 KQLPKKNCGECGTPTCLAFAMNLASGKASLDSCPYVSDAA 49
```

Figure S5. Membrane topology of RnfA, RnfD and RnfE predicted with the HMMTOP algorithm. The SGAT and SGTF motifs are highlighted. The topology was displayed using TOPO2.

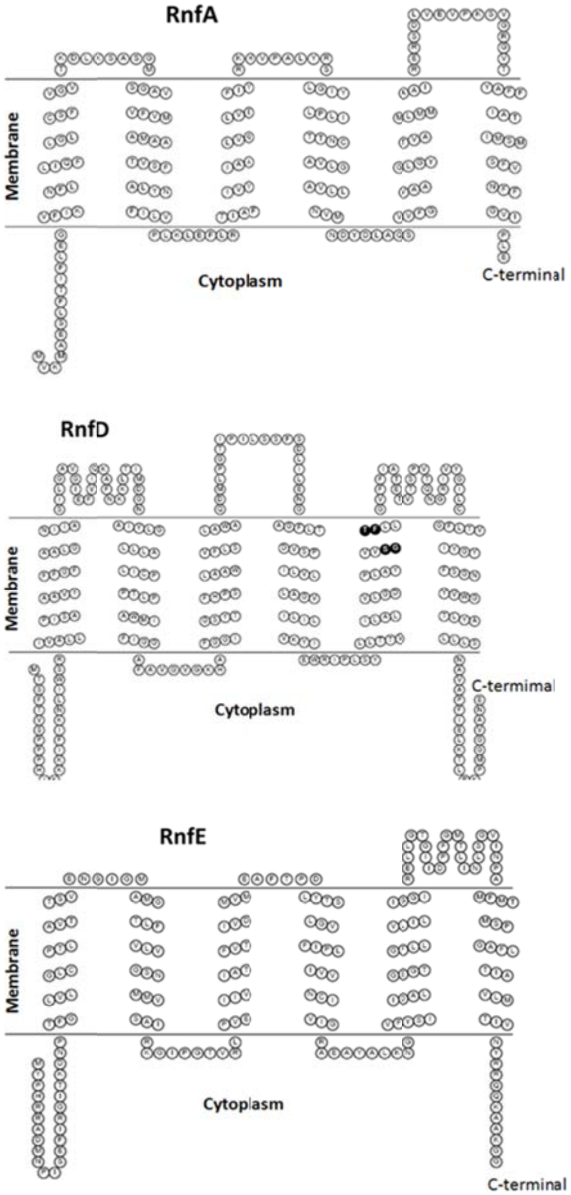

Supplement: File S1 — Supporting figures. Figure S1, SDS-PAGE of purified normal RnfG and variant RnfG-T166A from Methanosarcina acetivorans . Figure S2, Sequence alignment of RnfG from Methanosarcina acetivorans with NqrC from Vibrio cholerae . Figure S3, SDS-PAGE of RnfB from Methanosarcina acetivorans . Figure S4, Sequence comparisons of the RnfB subunit from Methanosarcina acetivorans . Figure S5, Membrane topology of RnfA, RnfD and RnfE predicted with the HMMTOP algorithm. (PDF) [file pone.0097966.s001.pdf]
